# Using a Triple Aim Approach to Implement “Less-is-More Together” and Smarter Medicine Strategies in an Interprofessional Outpatient Setting: Protocol for an Observational Study

**DOI:** 10.2196/13896

**Published:** 2019-07-18

**Authors:** Monique Lehky Hagen, René Julen, Pierre-Alain Buchs, Anne-Laure Kaufmann, Jean-Michel Gaspoz, Henk Verloo

**Affiliations:** 1 Valais Medical Society Sion Switzerland; 2 Pharmavalais Brig–Glis Switzerland; 3 Pharmavalais Sion Switzerland; 4 Institute of Health, HES-SO Valais-Wallis University of Applied Sciences and Arts Western Switzerland Sierre Switzerland; 5 Clinique des Grangettes Chêne-Bougeries Switzerland; 6 Health & Community Medicine Faculty of Medicine, School of Medicine University of Geneva Geneva Switzerland; 7 School of Health Sciences University of Applied Sciences and Arts Western Switzerland Valais (HES-SO Valais-Wallis) Sion Switzerland; 8 Service of Old Age Psychiatry University Hospital Lausanne Prilly Switzerland

**Keywords:** pharmacists, family physicians, prescription, deprescription, polypharmacy, evidence-based medicine

## Abstract

**Background:**

Increased awareness of the world’s problematic growing health care expenditure and health care shortages requires sustainable use of available resources. To promote cultural changes in medical mindsets, societies representing medical specialties have developed new Choosing Wisely strategies. The Valais Medical Society and the Valais Pharmacy Association have developed an interprofessional collaboration project entitled “Less-is-more Together-PPI” to analyze and optimize change management practices focusing on the prescription and deprescription of proton pump inhibitors (PPIs).

**Objective:**

This study aims to enhance interprofessional collaboration between physicians, pharmacists, and patients to optimize PPI use, avoid unnecessary treatments and improve therapeutic adherence to indicated therapies, and to analyze hindrances and facilitators to implementing interprofessional Less-is-more strategies in the field.

**Methods:**

Home-dwelling adults domiciled in Valais and prescribed PPIs in the last 6 months will be invited to participate in this observational study. The studied subpopulation will be constituted of consenting patients whose physicians and pharmacists also voluntarily agree to participate. The process of collecting, pooling, transmitting, evaluating, and protecting data has been validated by the Human Research Ethics Committee of the Canton Vaud.

**Results:**

The Primary Triple Aim outcome measures will be (1) population health: patient’s assessment of their own health, functional status, and disease burden using a monthly questionnaire for 6 months; Behavioral/physiological factors will be investigated using a final questionnaire at 6 months, (2) experience of care: assessment using a final questionnaire for participating patients, pharmacists and physicians, and an analysis of negative/positive experiences via 6 follow-up questionnaires, and (3) Per capita cost: participants’ fluctuating or decreasing PPI intake (number of pills/dosage) and an analysis of participants’ different categories following their medical prescription, in relation to possible bias effects on the overall drug intake of the population studied. Secondary outcomes will be participation rates; patient, physician, and pharmacist follow-up; and evaluations of participants' experiences and their perceived benefits, as well as whether the interprofessional process can be improved.

**Conclusions:**

This project seeks a deeper understanding of how Less-is-more and smarter-medicine strategies are perceived by patients and health care providers in their daily lives in a very specific context. It will reveal some of the hindrances to and facilitators for efficient cultural change toward a more sustainable health care system. The results will be useful to optimize and scale up further Choosing Wisely approaches.

**International Registered Report Identifier (IRRID):**

DERR1-10.2196/13896

## Introduction

There is growing awareness that the world’s ever-increasing health care expenditure and lack of global health care coverage will require a more sustainable use of available resources. With this in mind, Choosing Wisely strategies have been developed to promote a cultural shift in medical mindsets, mainly by societies of medical specialists. To date, little is known about the implementation and impact of these strategies [1,2]; however, there is nevertheless rising pressure to impose more sustainable health care strategies, including specific restrictions, coming from political and health insurance lobbies [3].

Choosing Wisely was an initiative of the American Board of Internal Medicine (ABIM), which sought greater dialogue between patients and physicians on avoiding unnecessary medical tests, treatments, and procedures [4,5]. The initiative was sparked by a 2010 *Perspective* article calling on American medical societies to identify 5 tests or treatments that were overused in their specialty and failed to provide any meaningful benefit to patients [6]. Some estimates have suggested that as much as 30% of all health care spending is wasted [7]. In 2012, the ABIM Foundation and Consumer Reports launched the Choosing Wisely campaign, inspired by the idea that professional societies and health care providers should take the lead in defining and motivating efforts to reduce the use of low-value care [6]. Since its launch, Choosing Wisely has grown rapidly and spread to 20 countries, with over 70 participating medical societies. Multiple national organizations, representing various medical specialties, have identified the tests or procedures that are commonly used in their field but whose necessity is questionable [2].

The latest report from the Organisation for Economic Co-operation and Development showed that the Swiss are among those who spend the most on health care [7]. In 2012, the Swiss Academy of Medical Sciences developed a roadmap on the theme of sustainable medicine [6]. In 2014, The Swiss Society of General Internal Medicine took the lead by introducing a program entitled Smarter Medicine—Choosing Wisely Switzerland [3]. A top-5 list overused in ambulatory general internal medicine was launched, which included a recommendation to avoid or discontinue proton pump inhibitors (PPIs) [3]. To give the Less-is-more Together approach a wider and not only medical audience, Choosing Wisely Switzerland’s leadership was transferred to a broader interprofessional alliance in 2017 [8]. *Less-is-more* is described as an invitation to recognize the potential risks of overuse of medical care that may result in harm rather than in better health [9]. Its ambition remains to move from a concept of maximum medicine to one of optimal medicine by relying on research and teaching grounded in evidence-based medicine. Faced with this context, the Valais Medical Society and the Valais Pharmacy Association launched a joint study to evaluate the extent to which interprofessional collaboration could affect the implementation of a smarter-medicine guideline in an outpatient setting under real-life conditions. The study focused on optimizing PPI use within the framework of the Smarter Medicine—Choosing Wisely Switzerland program [8].

### Why Should Proton Pump Inhibitor Use be Challenged?

The challenge of PPI prescription is that sometimes long-term intake is mandatory for medical reasons, but at other times short-term or on-demand-treatment would be sufficient. For various reasons, short-term treatments sometimes become long-term ones, but this situation deserves to be challenged [10]. PPIs reduce stomach acid production by inhibiting the H+/ K+-ATPase pump. They are indicated for the treatment of gastroesophageal reflux disease, esophagitis, peptic ulcer disease, Zollinger–Ellison syndrome, Helicobacter pylori eradication, secondary prevention of gastrointestinal bleeding under aspirin, and prevention of gastroduodenal lesions induced by nonsteroidal anti-inflammatory drugs [11,12]. First introduced in 1987, PPI use has increased significantly in the last 20 years, and they are now widely prescribed in Western countries, following specific recommendations and requirements [13]. Certain recommendations that aimed at older adults probably contributed to the increasing quantities of PPIs prescribed (around 10% per year in the 2000s) [10,14]. PPI treatments are generally considered to create few tolerance problems, but in recent years the scientific literature has described some side effects because of long-term use [15,16]. Studies have shown associations between PPIs and enteric infections [17,18], pneumonic pathologies [18,19], calcium malabsorption leading to an increased risk of osteoporotic fractures [20,21], and iron or vitamin B12 malabsorption causing anemia [18,21,22] and hyponatremia [23]. The use of PPIs for older adults has been associated with functional decline, more hospitalizations, and early death [24-26]. Another problem with prescribing PPIs, mainly among elderly polymedicated patients, is the existence of drug interactions that can lead to greater numbers of adverse effects of inactivated drugs (eg, citalopram) and, conversely, the decreased efficacy of prodrugs metabolized by this cytochrome [18,27,28]. PPIs may increase (eg, digoxin) or decrease (eg, ketoconazole) the absorption of certain drugs depending on gastric pH [28,29]. Despite the publication of multiple recommendations on good PPI prescribing, several studies report that they are poorly adhered to [30,31]. Several European studies have found a high frequency of inappropriate PPI prescriptions in hospitals and outpatient practices [32-34].

Using an interprofessional approach, these experiments will involve primary care physicians, pharmacists, and patients in canton Valais with a view to optimizing PPI prescription.

### The Observational Study: Less-is-More Together—Proton Pump Inhibitors in Valais

Challenged by the Smarter Medicine Program: Choosing Wisely Switzerland, the Medical Society of Valais and the Valais Pharmacy Association developed an observational study within the broader framework goal of optimizing the prescription and deprescription of PPIs among community-dwelling adults relying on interprofessional resources. This prescription/deprescription program aims to develop a more appropriate intake of PPIs based on the most recently published evidence-based guidelines [35] and, where possible, to move toward the deprescription of PPIs. The present project will focus on exploring the opportunities for increased collaboration between family physicians and community pharmacists, while also proactively integrating community-dwelling adult outpatients. The observational study framework will be built on the evidence-based PPI Bruyère Deprescribing guidelines [35]. Our observational study will investigate a simple community-engagement model to raise awareness of and promote the uptake of deprescribing initiatives, with the goal of scaling-up similar activities across the canton.

### Study Aims

The study’s primary aim is to enhance interprofessional collaboration between physicians, pharmacists, and patients to optimize PPI use, avoid unnecessary treatments, and improve therapeutic adherence where indicated. Secondary aims involve analyzing the factors hindering or facilitating the implementation of interprofessional Less-is-more Together strategies, drawing together feedback from patients and health care professionals and deciding how the impact of such projects can be measured efficiently.

## Methods

### Design

The Less-is-more Together—PPI study is framed as an observational study being the first step in a small-sample *plan-do-study-act* cycle, following the Institute of Health Improvement’s Triple Aim Initiative approach [36].

### Research Population

Home-dwelling adults in Valais who were prescribed any type of PPI in the last 6 months will be invited to participate in this observational study, if their community pharmacists and their primary health care physicians are willing to participate. The study’s final subpopulation will be made up of consenting patients who have been prescribed PPIs, for various medical indications, in the 6 months before the project’s initiation and whose physician and pharmacist both voluntarily agree to participate. To enable scientific follow-up and analysis of the outcomes of this particular smarter-medicine goal, patients were stratified according to known, evidence-based, deprescription strategies introduced in this framework by the Less-is-more Together—PPI project.

### Recruitment and Data Collection Procedure

Information on the project and the PPI guidelines of the Less-is-more Together—PPI project will be spread to the population by the local media but also specifically to individual members of the canton’s medical and pharmacy societies. Before participant recruitment and data collection, the study protocol is been approved by the Cantonal Research Ethics Committee, Vaud/Valais (CER-VD: 2018-00943). Written informed consent will be obtained from all participants before data collection commences, confidentiality will be ensured and preserved in all cases, and the study will also fulfil the provisions of the Declaration of Helsinki. Data collection will be organized into 2 phases: data collection from pharmacists’ electronic records of home-dwelling adults with a PPI prescription in the last 6 months and a prospective data collection.

In collaboration with participating primary care physicians and community pharmacists, electronic patient/client records will be searched for all the PPI prescriptions dispensed in the last 6 months. Each patient’s age, sex, place of residence, his pharmacist, and prescribing physician will be identified for use as the basic sample in the prospective study. Data will be extracted from eligible patients’ records according to procedural guidelines, entered into the database, and checked for plausibility and completeness. Participating pharmacies will send lists of eligible patients and their PPI prescriptions in the preceding 6 months to their respective primary health care physicians. The physicians will then apply the following procedure:

- Exclude patients who lack the faculty of judgment to participate in the study or patients with more than 1 pharmacist
- Group PPI users according to 1 of the 3 Bruyère categories [35]
- In case of changes to the PPI prescription during the study, the physician will communicate with the participating pharmacist (physician will send a new prescription by email and will adapt the PPI classification)
- Answer the end-of-project satisfaction questionnaire

Physicians will categorize the participating community-dwelling adult’s PPI indication [35] as follows:

1. Medical indication for regular prescription of PPIs at a minimum dose
2. Prescription with the aim of only taking PPIs “on demand”
3. Deprescription is possible, but the indication for possible dose reduction remains unclear
  - Decrease the PPI dose while maintaining a regular minimum dose intake (> 6 months)
  - Progressive decrease in prescribed PPI dose with the aim of stopping in the next few weeks (< 6 months)
  - Cessation of regular PPI intake and use only “on demand”
  - A complete cessation of the PPI prescription and intake

The participating physicians will be asked to categorize their patients based on the deprescription guidelines/flow chart published by *deprescribing.org* —the PPI deprescription algorithm [35].

### Prospective Data Collection

#### Sample

The convenience sample will be composed of eligible patients with a PPI prescription given by a primary care physician and followed up by participating pharmacists in the Valais region. To have sufficient statistical power, based on an 80% probability of not rejecting the null hypothesis (type-II error) and on reaching a statistical significance level of 0.05 (type-I error), a minimum of 500 patients will be required.

#### Recruitment

##### Physicians

The research team will contact all the primary health care physicians in the Valais Medical Society to request their collaboration in our research project. A flyer will explain the study’s aims, the participants involved, data collection procedures, and the researchers’ expectations for participants (Multimedia Appendix 1).

##### Pharmacists

The research team will also contact all the pharmacists in the Valais Pharmacy Association to request their collaboration. They will receive the same flyer. Participating pharmacists will extract the details of all the patients eligible for participation in the project from their databases. All patients who have been prescribed a PPI during the last 6 months, in agreement with their treating physicians (who also accept to participate in the study), will be listed and invited to participate. Patients will be contacted personally by the pharmacist and informed about the study. The patient will receive a comprehensive explanatory flyer and be invited to fill in the form to confirm their consent to participate (Multimedia Appendix 2). Attending physicians will be notified about participating patients and group them according to therapeutic categories (see above).

##### Patients

The physicians will prospectively recruit patients based on the retrospective analysis of electronic patient/client records by community pharmacists. Patients with a PPI prescription written by a participating primary health care physician will be invited to participate in the study. A patient-oriented flyer will explain the study’s aims, data collection procedures, and researchers’ expectations (Multimedia Appendix 2).

### Data Collection

#### Home-Dwelling Adult Patients

Data collection on PPI prescribing and deprescribing will be organized over 6 months. After consenting to participate in the study, home-dwelling adult patients will receive the first self-administered questionnaire on PPI-use and its effects on their clinical symptoms. Self-administered questionnaires 2 to 6—on PPI use, clinical symptoms, and prescription/deprescription—will be submitted to the participating patients monthly. The seventh and final questionnaire will assess patients’ level of satisfaction and the clinical benefits they perceived from this interprofessional practical-experimental project.

#### Primary Health Care Physicians and Pharmacists

Primary health care physicians and pharmacists will also receive a final questionnaire to assess their levels of satisfaction and the benefits they perceived from this interprofessional practical-experimental project.

### Data Collection Procedure and Ethical Considerations

Data collection, pooling, transmission, and evaluation processes will be done in conformity with data protection policies and have been validated by the Human Research Ethics Committee of the Canton Vaud. Data anonymization and confidentiality are guaranteed by a blinded coding process between pharmacists and the Haute école spécialisée de Suisse Occidentale, with specifically restricted access to the data. The first pooling of anonymized data is done at an administrative level by the Valais Pharmacy Association, and after a second coding it is transmitted to the RedCap system, where analysis takes place using an anonymized, standardized procedure.

All participants will receive oral and written information about the study before any data are collected. Participants’ written informed consent will be required to authorize primary care physicians and pharmacists to disclose their PPI medication prescriptions to the research team and to participate in this interprofessional study and collaboration project. Participants who refuse to give consent will be excluded from the project. Participants will be free to withdraw from the study at any time during data collection, without the need to provide any justification. In the event of revocation of consent, the law provides that data may still be used as long as they are subsequently anonymized. These clauses will be written into the information and consent form and will be discussed before each patient interview. After written consent, participants will be able to use electronic or paper questionnaires. Multimedia Appendices 1,2, and 3 present the data collection procedure.

### Data Analysis and Secure Storage

#### Data Analysis

Sociodemographic data, category distributions, and questionnaire data will be analyzed using descriptive statistics: distributions, frequencies, minimum, maximum, mean/median, SDs/interquartile ranges, and exact tests (chi-squared). Odds ratios/relative risks will be explored. Inferential statistics will be applied to analyze changes in categories and associations between sociodemographic data, categories, and questionnaires. *t* tests, analysis of variance, and repeated measurements will be applied where appropriate. An appropriate strategy will be applied to deal with missing data. The statistical level of significance will be set by considering the number of variables and the range of the databases. Data will be analyzed using the IBM Statistical Package for Social Sciences, version 25.0 (IBM Corp).

#### Data Storage

RedCap software will be used for data collection and the storage of sociodemographic data, participant data, and responses to the initial and final questionnaires completed by patients, pharmacists, and attending physicians. At each stage in the research, quality control will be maintained according to the standard quality criteria for quantitative research. The file linking participant identities to their identifying information will only be accessible by password to research team members (protection by RedCap). Participants' data will be stored in another file, also only accessible to participating pharmacists and research team members using a secure password. This file will only contain the identities and data analyzed in the study; these data will not contain any other information and they will be destroyed no later than 10 years after the end of data collection.

### Methods to Minimize Bias

All research team members will apply the same instructions and ensure that patients, pharmacists, and attending physicians receive the same explanations and instructions. Information sheets, consent forms, and questionnaires will be pretested on 3 patients who meet the inclusion criteria. On the participants' side, one possible bias is that of *social desirability*: patients may feel obligated toward persons associated with medical authority and seek to respond to what they see as an assessment of their adherence to treatment—responding with what they think is the right or desirable answer. The research team will be very attentive and caring to insist among the participants to be free to participate or not at the study. The oral and written information transmitted to the participants will place a particular emphasis on describing the researchers’ independence and nonjudgmental practices.

## Results

The Primary Triple Aim outcome measures will be as follows:

- Population health: continuous health and functional status and disease burden via a monthly questionnaire filled in by patients for 6 months; behavioral/physiological factors via a final questionnaire after 6 months:
  - Fluctuation, decrease, and increase in PPI prescribing and intake by category (1, 2, and 3)
  - Measurable impact of category 3 in comparison to all 3 categories as a group
  - Potential impact of the project on other aspects of health as perceived by the patient, pharmacist, and physician.
- Experience of care: via a final participant questionnaire (patients/pharmacists/doctors) and analysis of negative/positive experiences via the 6 follow-up questionnaires.
- Per capita cost: as it will be impossible to evaluate the real per capita cost in our context, this outcome will be analyzed mainly by evaluating the fluctuation or decrease in PPI intake (number of pills/dosage) by patients. The grouping of patients into different categories, according to their medical prescription, will be analyzed relative to possible bias effects on that population’s overall drug intake. Perceived benefits/negative side-effects will be analyzed via questionnaire answers and extrapolated as soft factors.

Secondary outcomes will be participation rates; patient, physician, and pharmacist follow-up; and evaluations of participants' experiences, the benefits they perceived, and how they think the process could be improved.

### Other Variables

Participants’ sociodemographic variables will be analyzed to describe the sample’s age, sex, geographic location (reference pharmacy’s urban or rural location), and mother tongue.

## Discussion

This project will help us to develop a deeper understanding of how Less-is-more Together and smarter-medicine strategies are perceived by patients and health care providers in their daily lives in a very specific setting. It will reveal the hindrances and facilitators to efficient cultural changes to a more sustainable health care system—factors which can be taken advantage of to scale-up the application of further Choosing Wisely approaches in Switzerland. This project uses a novel approach implying more active involvement by patients and their pharmacists in the implementation of a more responsible and interprofessional approach to prescription. The analysis of different partners’ perceptions will help us to determine whether and how this reinforced interprofessional involvement should be fostered. Evaluating this practical experiment’s interprofessional approach to implementing a Less-is-more Together-type strategy could lead to the development of an interprofessional example of good practice, which could be used to promote implementation of other Choosing Wisely Switzerland’s initiatives.

## Appendix

Multimedia Appendix 1 Data collection strategy. CER-VD: Cantonal Research Ethics Committee, Vaud/Valais; HES-SO: Haute école spécialisée de Suisse occidentale; ID: identity document; PPI: proton pump inhibitors; SMV: Medical Society of Valais; UAS: University of Applied Sciences.

Multimedia Appendix 2 Data collection strategy—part II. ID: identity document.

Multimedia Appendix 3 Project planning—Less-is-more Together—proton pump inhibitor.

## Abbreviations

ABIM: American Board of Internal Medicine
PPI: proton pump inhibitor
