# Supplementary figures and images for: Using a Triple Aim Approach to Implement “Less-is-More Together” and Smarter Medicine Strategies in an Interprofessional Outpatient Setting: Protocol for an Observational Study
Source: JMIR Res Protoc. 2019 Jul 18;8(7):e13896. doi: 10.2196/13896 (PMC6670276; doi:10.2196/13896)

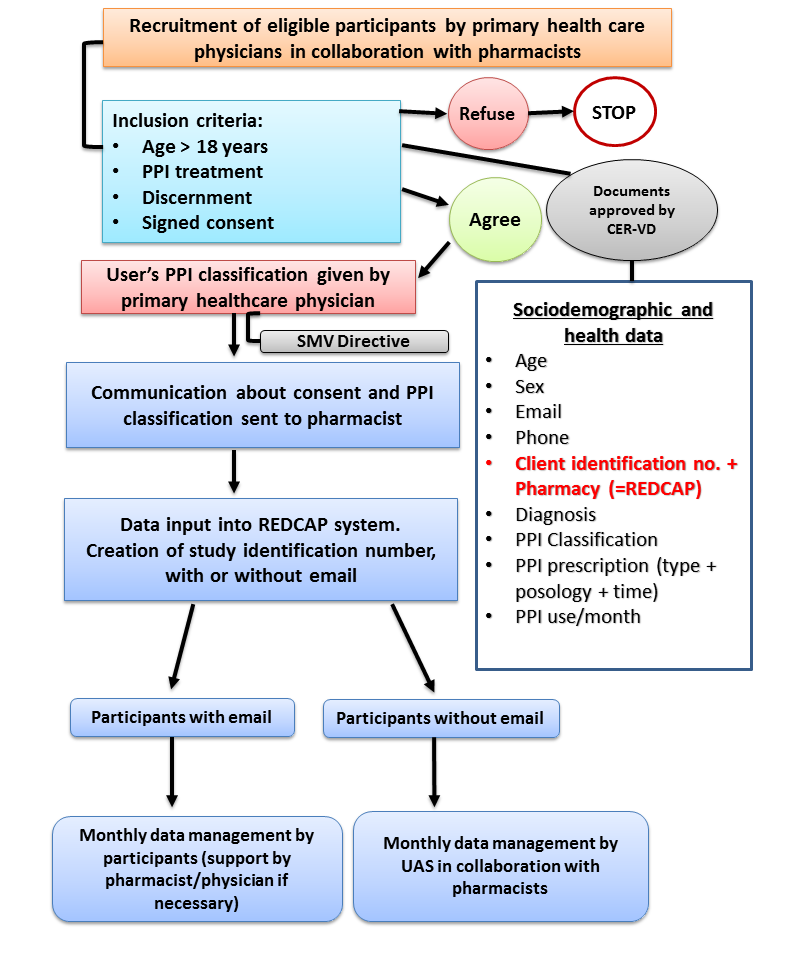

Supplement: Multimedia Appendix 1 [file resprot_v8i7e13896_app1.PNG]

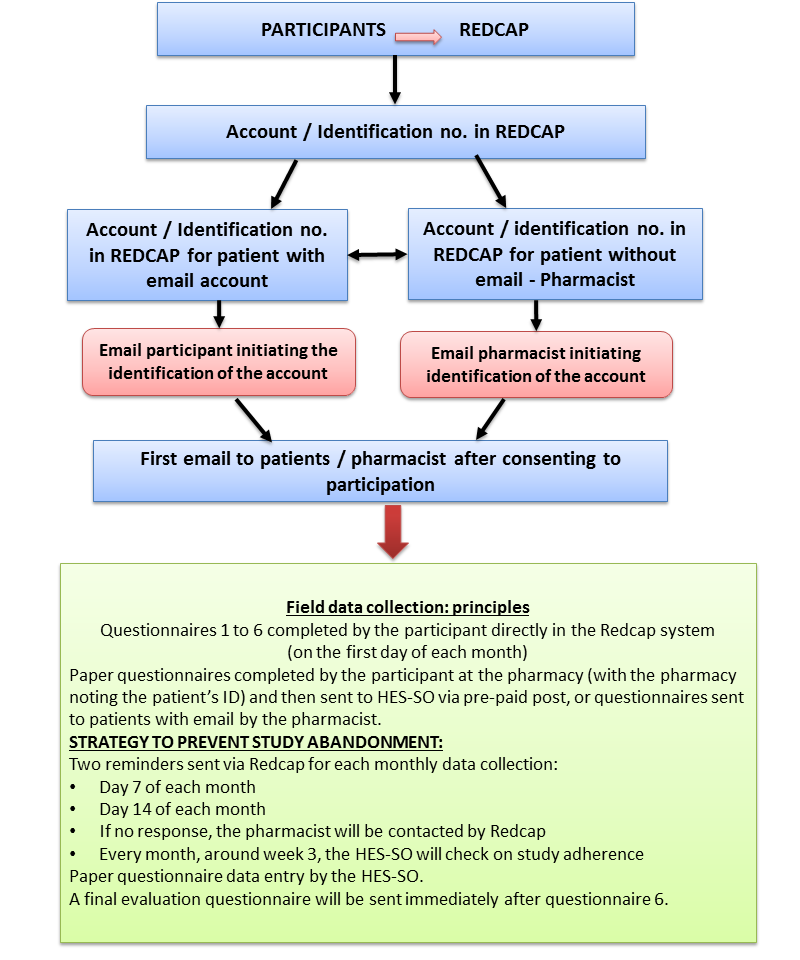

Supplement: Multimedia Appendix 2 [file resprot_v8i7e13896_app2.PNG]

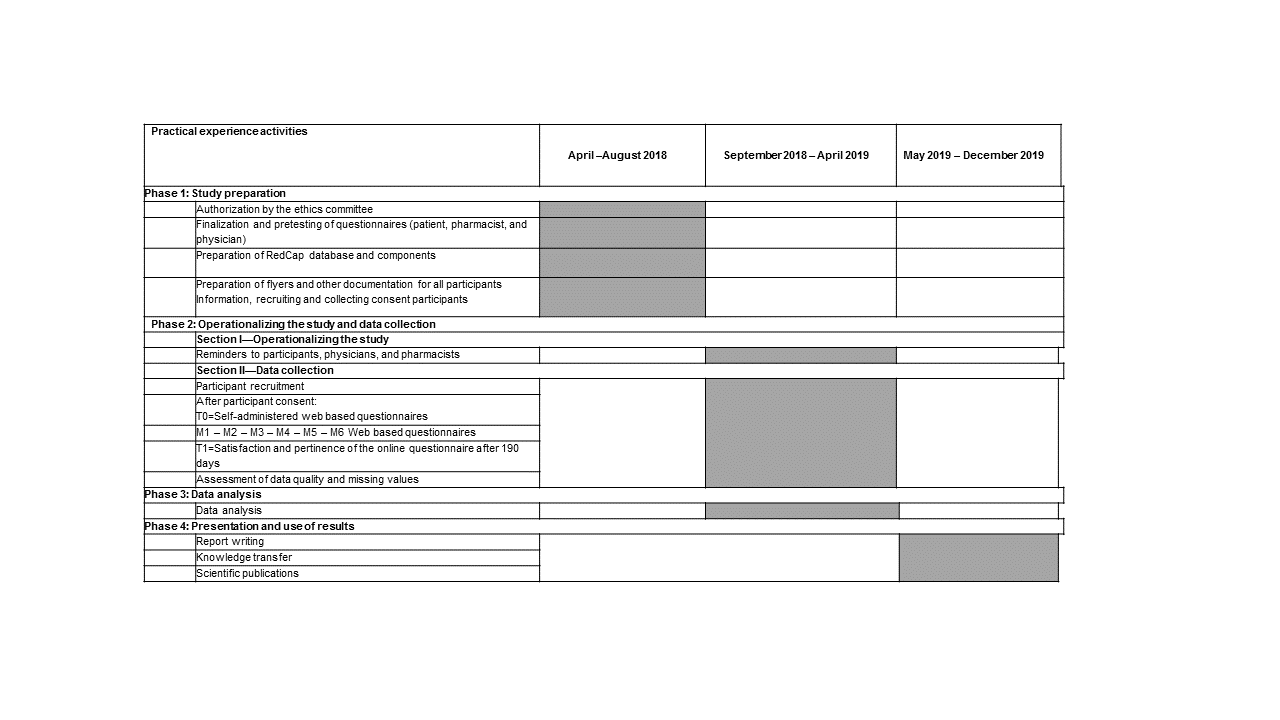

Supplement: Multimedia Appendix 3 [file resprot_v8i7e13896_app3.PNG]
